# Association between parental support for physical activity and fundamental movement skills in children

**DOI:** 10.3389/fpsyg.2026.1808648

**Published:** 2026-05-21

**Authors:** Yijun Zheng, Chuifeng Kong, Kai Li

**Affiliations:** 1 Guangdong Pharmaceutical University, Guangzhou, Guangdong, China; 2 School of Physical Education, Shanghai University of Sport, Shanghai, China; 3 College of Sports Industry and Leisure, Nanjing Sport Institute, Nanjing, Jiangsu, China

**Keywords:** children, fundamental movement skills, Macau, parental support, physical activity

## Abstract

**Objective:**

To assess the level of fundamental movement skills (FMS) among children aged 6–10 years in Macau and to examine the associations between parental support for physical activity and children's FMS.

**Methods:**

A total of 270 children aged 6–10 years were recruited, and 243 valid samples were included in the final analysis. Fundamental movement skills were assessed using the test of gross motor development, second edition (TGMD-2), and parental support was measured using the activity support scale for multiple groups (ACTS-MG). Descriptive statistics, independent samples *t*-tests, one-way ANOVA, and hierarchical multiple linear regression analyses were conducted to examine differences and associations.

**Results:**

The mean total FMS score was 66.72, with locomotor skills outperforming object control skills across all age groups. FMS scores increased significantly with age (*p* < 0.01). Parental support levels were generally moderate to high, although parental modeling and community resource utilization showed relatively lower scores. Hierarchical regression analyses indicated that logistical support was significantly associated with locomotor skills (β = 0.311, *p* < 0.01), object control skills (β = 0.117, *p* < 0.05), and total FMS (β = 0.288, *p* < 0.01), while community resource utilization was significantly associated with locomotor skills (β = 0.253, *p* < 0.01) and total FMS (β = 0.204, *p* < 0.05).

**Conclusion:**

Children aged 6–10 years in Macau demonstrated relatively low levels of fundamental movement skills and an imbalance in skill development, with locomotor skills outperforming object control skills. Parental logistical support and community resource utilization were positively associated with children's FMS, highlighting the importance of strengthening family support and improving community-based physical activity environments to promote balanced motor development in children.

## 1 Introduction

Fundamental movement skills (FMS), which mainly include locomotor skills and object control skills (Ulrich, 2000), are considered the foundation for children's participation in physical activity and the development of lifelong exercise habits (Stodden et al., 2008; Robinson et al., 2015). Childhood is a critical period for the development of FMS, and a high level of motor competence not only promotes participation in physical activity but is also associated with improved physical fitness, health outcomes, and psychological development (Robinson et al., 2015; Barnett et al., 2016; Bolger et al., 2021). Previous studies have shown that insufficient development of FMS may limit children's engagement in physical activity and increase the risk of sedentary behavior and related health problems (Stodden et al., 2008; Barnett et al., 2016). Therefore, understanding the factors influencing children's FMS development is essential for promoting healthy growth.

In recent years, research has increasingly shifted from focusing solely on individual physiological factors to examining the influence of social and environmental determinants on children's motor development (Barnett et al., 2013; He et al., 2024). Ecological models of motor development suggest that motor competence is shaped not only by biological maturation but also by practice opportunities, family support, and environmental conditions (Stodden et al., 2008; Robinson et al., 2015). Among these factors, parental support plays a particularly important role in shaping children's physical activity behaviors (Liu et al., 2024). Parental support was conceptualized as a multidimensional construct including logistical facilitation, behavioral modeling, utilization of activity resources, and regulation of sedentary behavior (Trost et al., 2003; Beets et al., 2010). Parents may increase children's opportunities for movement practice by providing logistical support, participating in activities, serving as role models, and facilitating access to physical activity environments (Flynn et al., 2024). Previous studies have demonstrated significant associations between parental support and children's physical activity levels (Davison et al., 2011; Lampard et al., 2016), and different types of parental support may exert distinct effects on children's behavior (Pyper et al., 2016). Parental support for children's physical activities may create opportunities for the practice of sports skills, and by influencing the important factors of the external support environment for FMS, it is beneficial for improving the level of motor development (Stodden et al., 2008). However, most existing studies have focused on physical activity participation rather than motor skill development, and empirical research examining the relationship between parental support and FMS remains limited (Beets et al., 2010; Su et al., 2022).

In addition to family influences, community environments also play an important role in shaping children's physical activity opportunities (Carver et al., 2023; Smith et al., 2022). Access to sports facilities, availability of safe play spaces, and participation opportunities in organized activities may increase children's movement experiences and contribute to motor skill development (Barnett et al., 2016). Furthermore, previous research has suggested that different domains of FMS may respond differently to environmental influences, with object control skills often requiring more structured practice and instruction, whereas locomotor skills may develop more naturally through daily activities (Bolger et al., 2021; He et al., 2024; Logan et al., 2012). While community environments have been widely recognized as important determinants of children's physical activity and motor development, these influences may vary considerably across different urban contexts. In densely populated Asian cities, factors such as limited access to open play spaces, high academic demands, and constrained opportunities for unstructured outdoor activity may further restrict children's movement experiences (Ding et al., 2011; Bishop et al., 2020; Bao et al., 2021).

Within this broader context, Macau represents a particularly relevant setting for examining these issues. As one of the most densely populated regions in the world, Macau is characterized by limited recreational space, high levels of urbanization, and demanding educational expectations (Wang et al., 2025; Li et al., 2024). These socio-environmental conditions may shape both children's opportunities for movement practice and the ways in which parents support their children's physical activity. Previous research has indicated that family participation in sports in Macau remains relatively low (Wu et al., 2009). Despite the importance of both family and community factors, empirical evidence examining the combined influence of parental support and community resources on different domains of FMS remains limited, particularly in Asian populations.

Therefore, the purpose of this study was to assess the level of FMS among children aged 6–10 years in Macau and to examine the relationship between parental support for physical activity and children's FMS. In addition, this study explored how different types of parental support were associated with locomotor and object control skills, with the aim of providing evidence to inform family-based interventions and community physical activity promotion strategies. Based on previous literature and theoretical considerations, it was hypothesized that different types of parental support would be differentially associated with fundamental movement skill domains. Logistical support and community resource utilization were expected to show stronger associations with locomotor skills, whereas object control skills were expected to be less influenced by environmental support alone.

## 2 Methods

### 2.1 Participants

A total of 270 children aged 6–10 years were recruited in Macau using a convenience sampling approach. Participants were recruited through local primary schools based on accessibility and willingness to participate. All eligible children within the target age range were invited to participate, and inclusion was based on the return of signed informed consent forms from parents or guardians. An *a priori* sample size estimation was conducted using G^*^Power 3.1 for multiple linear regression. Assuming a medium effect size (*f*^2^ = 0.15), an alpha level of 0.05, a statistical power of 0.80, and up to five predictors, the minimum required sample size was 92. Therefore, the final valid sample size in this study was considered adequate for the planned analyses.

### 2.2 Measures

#### 2.2.1 Fundamental movement skills

Fundamental movement skills were assessed using the Test of Gross Motor Development, Second Edition (TGMD-2) (Ulrich, 2000). This assessment tool is designed for children aged 3–10 years and has been demonstrated to have good reliability and validity among Chinese children (Li and Diao, 2013).

The TGMD-2 includes two subtests: locomotor skills and object control skills. Locomotor skills include running, galloping, hopping, leaping, horizontal jumping, and sliding. Object control skills include striking a ball, stationary dribbling, catching, kicking, overhand throwing, and underhand rolling, resulting in a total of 12 skills. Before testing, all examiners participated in three training sessions to ensure a thorough understanding of the testing procedures and protocols. The testing procedure consisted of two steps. First, a trained instructor provided a standardized silent demonstration of each skill. Second, during formal testing, each child performed two consecutive trials of each skill. A video camera was positioned to record each child's performance throughout the testing process. All TGMD-2 assessments were conducted in indoor gymnasium settings within the participating schools to ensure a consistent and safe testing environment. Children completed the tests during regular school hours in a comfortable, distraction-free, and low-pressure setting to minimize external influences on performance. Testing conditions were standardized as much as possible across sessions.

After testing, two trained raters independently scored the video recordings. Each skill was evaluated based on 3–5 performance criteria. The maximum total score was 96 points, including 48 points for locomotor skills and 48 points for object control skills. Inter-rater reliability assessed using Pearson correlation coefficients ranged from *r* = 0.86 to 0.98 (*p* < 0.01), indicating good scoring reliability.

#### 2.2.2 Parental support

Parental support was assessed using the parent-report version of the Activity Support Scale for Multiple Groups (ACTS-MG), rather than an instrument specifically designed to measure support for motor skill learning (Davison et al., 2011; Lampard et al., 2016). This construct was conceptualized as an external supportive environmental factor, which may influence children's motor development indirectly by increasing opportunities for physical activity participation, structured practice, and engagement in activity-supportive environments.

The parent-report approach was considered appropriate given the young age of the participants, as children aged 6–10 years may have difficulty accurately recalling and reporting parental support behaviors. Therefore, parent-report measures may provide a more feasible and informative assessment in this age group. The ACTS-MG consists of four dimensions and 15 items (see Table 1).

**Table 1:** Dimensions, conceptual definitions, and measurement items of parental support assessed by the ACTS-MG.

Dimension	Conceptual definition	Items
Logistical support	Parents provide opportunities and support for children's participation in physical activity	• I enroll my child in sports teams and clubs such as soccer, basketball, and dance. • I take my child to places where he/she can be active. • I watch my child play sports or participate in other activities such as martial arts or dance.
Parental modeling	Parents encourage children to participate in physical activity by serving as role models	• We have family outings that include physical activity (such going for a walk, bike riding, or ice skating). • I try to include my child when I do something active. • I encourage my child to be physically active by leading by example (by role modeling). • I exercise or am physically active on a regular basis. • I enjoy exercise and physical activity.
Community resource utilization	Parents facilitate children's opportunities for physical activity within the community	• I encourage my child to use resources in our neighborhood to be active (such as the park and the school). • I enroll my child in community-based programs (such as Girls and Boys Club, YMCA) where he/she can be active. • I find ways for my child to be active when school is out by, for example, enrolling him/her in summer camp and after school programs. • I encourage my child to walk or ride his/her bike in our neighborhood if it is safe and appropriate for his/her age.
Restriction of sedentary behavior	Parents limit children's sedentary behaviors	• I limit how long my child plays video games (including playstation, Xbox, and gameboys). • I limit how long my child can watch TV or DVDs each day (including educational and non-educational programs). • I limit how long my child can use the computer for things other than homework (such as playing computer games and surfing the internet).

All items were rated on a 4-point Likert scale: 4 = strongly agree, 3 = agree, 2 = disagree, and 1 = strongly disagree. Scores were summed to obtain subscale scores for each type of parental support. The ACTS-MG has been widely used internationally and demonstrated good reliability in previous studies (Lampard et al., 2016; Chen and Dai, 2016). In the present study, test–retest reliability was assessed with 115 parents over a 2-week interval, yielding reliability coefficients ranging from 0.534 to 0.955. Although some subscales demonstrated relatively lower coefficients, values above 0.50 are generally considered indicative of moderate reliability (Koo and Li, 2016). Internal consistency reliability was high, with a Cronbach's α of 0.928, and subscale α values ranging from 0.764 to 0.942, indicating good reliability (Wang et al., 2025).

### 2.3 Procedure

A sample of 270 children aged 6–10 years was recruited in Macau. First, informed consent forms (bearing the school seal) and parent questionnaires were distributed to the children, who delivered them to their primary caregivers. The informed consent form included statements regarding voluntary participation, confidentiality of information, and the use of data for scientific research. All primary caregivers signed the informed consent forms and completed the questionnaires, which were returned to teachers through the children. Second, trained examiners organized TGMD-2 testing during physical education classes, and all children completed the assessment. Finally, questionnaires with substantial missing data, invalid responses, or participants outside the target age range were excluded, as were invalid test data. A total of 243 valid samples were retained for analysis.

### 2.4 Statistical analysis

The data were statistically analyzed using SPSS 26.0 (IBM Corp., Armonk, NY, United States), with the significance level set at *p* < 0.05. Descriptive statistics (mean and standard deviation) were used to summarize children's FMS and parental support scores. Independent samples *t*-tests were used to examine differences in FMS and parental support by gender, and one-way analysis of variance (ANOVA) was used to examine differences across age groups. Finally, hierarchical multiple linear regression analyses using a stepwise entry method were conducted to examine the relationships between parental support and children's FMS while controlling for age.

## 3 Results

### 3.1 Participant characteristics

Among the 270 participants, a total of 243 children provided complete and valid data, including 140 boys (51.9%) and 103 girls (38.1%), with a mean age of 7.91 ± 1.33 years. For the ACTS-MG questionnaire, the respondents were primarily parents (96.7%), including 165 mothers (67.9%) and 68 fathers (28.2%).

### 3.2 Fundamental movement skills of children

**Figure 1:**
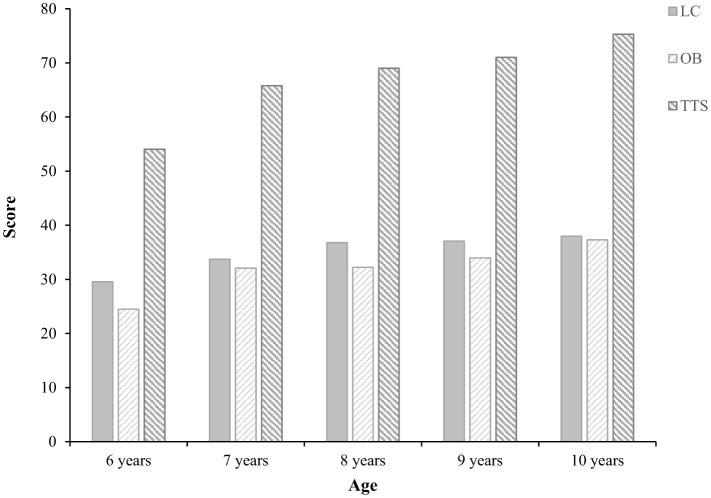
Trend of changes in children's fundamental movement skills scores. LC, locomotor skills; OB, object control skills; TTS, total test score.

As shown in Table 2, the mean total score of children's FMS was 66.72, while the mean scores for locomotor skills and object control skills were 34.96 and 31.76, respectively. Figure 1 shows that scores for locomotor skills, object control skills, and total FMS increased progressively with age among children aged 6–10 years. In all age groups, locomotor skill scores were higher than object control skill scores. The results of the difference analysis indicated that all FMS scores showed significant differences across age groups (*P* < 0.01). A significant gender difference was found in locomotor skills, whereas no significant gender differences were observed in object control skills or total FMS scores.

**Table 2:** Results of children's fundamental movement skills and parental support tests (*x* ± *s*).

Variable	*N*	Fundamental movement skills			Logistic support	Parental support		
		Locomotor skills	Object control skills	Total test score		PA model	Community resource utilization	Restrictions on sedentary behavior
6 years	48	29.56 ± 6.89	24.48 ± 6.35	54.04 ± 11.57	9.08 ± 1.41	13.85 ± 2.75	11.42 ± 2.27	9.48 ± 1.40
7 years	49	33.71 ± 6.05	32.08 ± 6.45	65.80 ± 10.30	9.14 ± 1.80	14.00 ± 3.20	11.61 ± 2.24	9.73 ± 1.56
8 years	55	36.78 ± 5.20	32.22 ± 5.63	69.00 ± 8.91	8.95 ± 1.57	13.71 ± 2.84	11.62 ± 2.01	9.47 ± 1.64
9 years	59	37.05 ± 6.43	33.97 ± 5.54	71.02 ± 9.91	8.92 ± 1.90	14.00 ± 2.81	11.24 ± 2.27	9.56 ± 1.84
10 years	32	37.97 ± 6.49	37.31 ± 3.55	75.28 ± 8.16	9.16 ± 1.87	14.59 ± 3.01	12.03 ±± 2.02	9.69 ± 1.60
total	243	34.96 ± 6.85	31.76 ± 6.93	66.72 ± 12.01	9.03 ± 1.70	13.98 ± 2.90	11.54 ± 2.17	9.58 ± 1.62
age		< 0.001	< 0.001	< 0.001	0.934	0.738	0.551	0.907
gender		0.023	0.970	0.222	0.976	0.975	0.078	0.540

### 3.3 Parental support

The results of the parental support survey showed that the midpoint of the ACTS-MG scale was 2.5. The mean scores of all dimensions were higher than this midpoint, including restriction of sedentary behavior (*M* = 3.19, SD = 0.54), logistical support (*M* = 3.01, SD = 0.57), community resource utilization (*M* = 2.89, SD = 0.54), and parental modeling (*M* = 2.80, SD = 0.58). These findings indicate that parents in Macau generally reported relatively high levels of support for children's physical activity; however, the levels of community resource utilization and parental modeling were relatively lower compared with other types of support. In addition, Table 2 shows that no significant differences in parental support were found across gender or age groups, suggesting that parental support behaviors remained relatively stable and showed only minor fluctuations across age.

### 3.4 Relationship between parental support and fundamental movement skills in children

Hierarchical multiple linear regression analyses were conducted. Age was entered in Model 1 as a control variable. Parental support variables were entered using a stepwise method in Model 2 and Model 3, with only significant predictors retained in the final models. As shown in Table 3, for locomotor skills, Model 1 indicated that age had a significant effect, explaining 16.2% of the variance. After controlling for age, Model 2 included logistical support and showed that logistical support was significantly positively associated with locomotor skills (β = 0.124, *p* < 0.05), and the explained variance increased to 17.4%.

**Table 3:** Regression analysis of parental support and fundamental movement skills for children.

Dependent variable	Model	Independent variable	*R*	*R* ^2^	$R_{\text{adj}}^{2}$	*B*	SD	β
Locomotor skills	1	Age	0.407	0.166	0.162	2.101	0.304	0.407^**^
	2	Age	0.425	0.181	0.174	2.112	0.302	0.409^**^
		Logistic support				0.499	0.235	0.124^*^
	3	Age	0.458	0.210	0.200	2.169	0.298	0.420^**^
		Logistic support				1.251	0.343	0.311^**^
		Community resource utilization				0.799	0.269	0.253^**^
Object control skills	1	Age	0.526	0.276	0.273	2.743	0.286	0.526^**^
			0.538	0.290	0.284			
	2	Age				2.754	0.284	0.528^**^
		Logistic support				0.475	0.221	0.117^*^
Total test score	1	Age	0.535	0.286	0.283	4.844	0.493	0.535^**^
	2	Age	0.553	0.305	0.300	4.866	0.487	0.538^**^
		Logistic support				0.974	0.379	0.138^*^
	3	Age	0.570	0.324	0.316	4.946	0.482	0.546^**^
		Logistic support				2.034	0.556	0.288^**^
		Community resource utilization				1.126	0.436	0.204^*^

**Correlation is significant at the 0.01 level (two-tailed). ^*^Correlation is significant at the 0.05 level (two-tailed).

Age was entered in Model 1. Parental support variables were entered stepwise in Models 2 and 3, with only significant predictors retained.

Further analysis in Model 3 included both logistical support and community resource utilization. The results showed that logistical support (β = 0.311, *p* < 0.01) and community resource utilization (β = 0.253, *p* < 0.01) were both significantly positively associated with locomotor skills, and the explained variance increased to 20.0%.

For object control skills, Model 1 showed that age had a significant effect, explaining 27.3% of the variance. After controlling for age, Model 2 indicated that logistical support was significantly positively associated with object control skills (β = 0.117, *p* < 0.05), and the explained variance increased to 28.4%.

For total FMS, Model 1 showed that age had a significant effect, explaining 28.3% of the variance. After controlling for age, Model 2 indicated that logistical support was significantly positively associated with total FMS (β = 0.138, *p* < 0.05), and the explained variance increased to 30.0%.

Further analysis in Model 3 showed that logistical support (β = 0.288, *p* < 0.01) and community resource utilization (β = 0.204, *p* < 0.05) were both significantly positively associated with total FMS, and the explained variance increased to 31.6%.

## 4 Discussion

This study investigated the influence of parental support for physical activity on FMS among children aged 6–10 years and compared the findings with relevant domestic and international studies. The results indicated that parental support for physical activity was positively associated with children's FMS. Specifically, logistical support and community resource utilization were significantly associated with children's total FMS scores and locomotor skills, whereas only logistical support showed a significant positive association with object control skills.

### 4.1 Children's fundamental movement skills

The findings of the present study indicated that the overall level of FMS among children aged 6–10 years in Macau was relatively modest. According to the normative data of the TGMD-2 (Ulrich, 2000), the average total scores observed in the current sample were comparatively lower, particularly in the domain of object control skills. Consistent with the present findings, previous research conducted in Macau has also reported that children's overall fundamental movement skill competence tended to be at a moderate or developmentally insufficient level (Xin et al., 2019). This developmental pattern is in line with evidence reported in other densely populated Asian urban contexts. For example, a study involving Hong Kong Chinese children found that more than 40% of participants were classified as having low or below-average FMS levels (Wong and Cheung, 2006), and similar trends have been observed among school-aged children in mainland China (Li and Diao, 2013), providing a culturally relevant regional benchmark. Evidence from a global systematic review based on TGMD-2 studies suggests that children in many countries and regions tend to demonstrate FMS levels ranging from below average to average relative to U.S. norms, indicating that insufficient motor skill development is a common phenomenon influenced by environmental and educational opportunities (Bolger et al., 2021).

In addition, children in Macau exhibited a clear imbalance in skill development, with locomotor skills outperforming object control skills. This finding is consistent with previous international studies. Systematic reviews have shown that children generally acquire locomotor skills such as running and jumping more easily, whereas the development of object control skills, such as throwing and catching, tends to lag behind (Bolger et al., 2021). This difference may be related to the way children acquire movement experiences. On the one hand, locomotor skills are common in daily life, and children can obtain frequent and spontaneous practice opportunities through play, outdoor activities, and daily mobility, making these skills easier to master (Stodden et al., 2008). On the other hand, object control skills often require specific equipment, appropriate space, peer interaction, and structured instruction (Stodden et al., 2008). When school-based physical education or family support is insufficient, children may have fewer opportunities to practice these skills, thereby limiting their development. Furthermore, object control skills rely more heavily on perceptual–motor integration, hand–eye coordination, and technical instruction, making their learning process more complex than that of locomotor skills (Stodden et al., 2008). Therefore, these skills are more susceptible to the influence of teaching environments and social support. Overall, insufficient practice opportunities and limited instructional support may be important factors contributing to the relatively lower object control skill performance observed in Macau children.

### 4.2 Parental support

The results showed that parents generally paid considerable attention to supporting children's physical activity; however, support in the form of role modeling and community resource utilization was relatively low. Similar patterns have been reported in previous studies, where parents were more likely to support children through encouragement and accompaniment, while their own participation in physical activity and proactive use of community resources were less frequent (Lampard et al., 2016,?).

Several factors may explain this phenomenon. Parents may have limited leisure time due to demanding work schedules, weaker personal exercise habits, and insufficient access to or awareness of community sports resources, all of which may restrict the provision of modeling and environmental support (Beets et al., 2010). In addition, compared with studies conducted in other countries using the same measurement scale, the overall level of parental support in this study appeared to be relatively lower (Davison et al., 2011). This may be related to the unique economic, cultural, and geographic characteristics of Macau. Rapid economic development and heavy workloads may reduce parents' available time for physical activity and family interaction, limiting their ability to serve as active role models for their children (Wu et al., 2009). In addition, strong academic expectations and school-related pressures in Macau may further encourage parents to prioritize structured schedules and logistical arrangements over time-intensive forms of engagement such as role modeling (Zhang et al., 2020; Padmapriya et al., 2024). Furthermore, as one of the most densely populated regions in the world, Macau has relatively limited community space for children's physical activity, and parental safety concerns may further reduce children's outdoor activity time (Pyper et al., 2016).

In addition, no significant differences in parental support were found across children's age groups or between genders, suggesting that parental support behaviors tend to remain relatively stable and may reflect general parenting styles rather than child-specific differences.

### 4.3 The relationship between parental support and children's fundamental movement skills

This study found that logistical support and community resource utilization were significantly associated with children's total FMS and locomotor skills, whereas only logistical support showed a significant association with object control skills. These findings indicate that family and environmental support play an important role in children's motor skill development. Logistical support typically involves providing time, transportation, equipment, and opportunities for participation in physical activities (Lampard et al., 2016). Such support may facilitate repeated engagement in skill-related activities and offer children access to environments in which FMS can be practiced and refined, thereby promoting skill acquisition and consolidation (Stodden et al., 2008). Previous studies have similarly emphasized that practice opportunities and supportive environments are key external determinants of motor competence (Stodden et al., 2008; Barnett et al., 2016).

Notably, parental behavioral modeling showed relatively weak associations with children's FMS, whereas logistical support—such as providing transportation to sports venues—emerged as a more salient predictor (Hosokawa et al., 2023). This pattern suggests that, within the context of Macau's high-density urban environment, children's opportunities for physical activity may be more strongly constrained by accessibility and environmental factors than by observational learning alone. In such settings, parental support that facilitates access—such as providing transportation, enrolling children in structured programs, and creating opportunities for participation—may play a critical “gatekeeping” role (Hosokawa et al., 2023). Even when parents themselves are physically active, children may have limited opportunities to engage in skill-related activities without this form of active facilitation. Therefore, in resource-constrained urban environments, the “facilitator” role of parents may outweigh the “model” role in supporting children's motor skill development (Pelyvás and Kovács, 2025; Menescardi and Estevan, 2021).

Further analysis showed that community resource utilization significantly influenced locomotor skills and total FMS scores but had no significant effect on object control skills. This may be explained by differences in how various motor skills are acquired. Locomotor skills such as running and jumping can often be practiced naturally in open environments such as parks and playgrounds; therefore, access to community sports facilities and activity spaces may directly promote their development (Barnett et al., 2016). In contrast, object control skills such as throwing and catching typically require structured practice and technical feedback, and reliance solely on community environments may not be sufficient to produce measurable improvements. In addition, public activity spaces in Macau may be relatively small and crowded, which may limit the safe practice of object control skills such as throwing and catching (Wang et al., 2025). In such environments, locomotor activities such as running and jumping may be more feasible, as they require less space and pose fewer safety constraints. Previous research has also indicated that different domains of FMS vary in their sensitivity to environmental and instructional support, with object control skills being more dependent on structured guidance (He et al., 2024; Logan et al., 2012).

These contextual constraints are not unique to Macau but are also observed in other high-density Asian cities such as Hong Kong and Singapore. For example, limited residential space and restricted access to private play areas are common in both Macau and Hong Kong, increasing reliance on public facilities for children's physical activity (Zhang et al., 2020; Sobko et al., 2017). In addition, strong academic expectations—often described in Singapore as a “kiasu” culture—may prioritize academic achievement over physical activity, thereby reducing time available for movement-related experiences (Strugnell et al., 2014). Furthermore, dual-income family structures are prevalent across these regions, which may influence how children's physical activity is organized and supported within the family. These similarities suggest that the present findings may have broader relevance to other high-density Asian contexts with comparable socio-environmental constraints.

Moreover, age was found to be a significant predictor of all domains of motor skills, indicating that children's motor competence improves naturally with age and accumulated movement experience (Ulrich, 2000; Bolger et al., 2021). However, even after controlling for age, logistical support and community resource utilization significantly increased the explanatory power of the regression models, suggesting that family and environmental support not only influence activity participation but may also contribute to the process of motor skill acquisition and development. These findings further support the ecological model of motor development, which proposes that motor competence results from the interaction of biological maturation, practice opportunities, and social environments (Stodden et al., 2008). Therefore, promoting children's FMS requires sustained family logistical support, improved accessibility and utilization of community physical activity resources, and targeted instruction and structured practice opportunities, particularly for object control skills, to achieve balanced motor development.

In summary, promoting the development of children's FMS requires sustained family logistical support, improved accessibility and utilization of community-based physical activity resources, and more targeted instruction and practice opportunities, particularly for object control skills, in order to achieve balanced skill development. These findings further highlight the importance of strengthening family logistical support and enhancing community physical activity environments to facilitate children's FMS.

## 5 Limitations and implications

This study has several limitations that should be considered when interpreting the findings. First, the cross-sectional design prevents causal inference between parental support and children's FMS. Second, parental support was assessed using a self-report questionnaire, which may be subject to recall or social desirability bias. Although appropriate for young children, this approach still has inherent limitations. Third, the sample was drawn from a specific regional population, which may limit the generalizability of the findings. Fourth, participants were recruited using a convenience sampling approach, which may introduce selection bias and limit the representativeness of the sample and the generalizability of the findings.

Despite these limitations, the present study provides meaningful implications for research and practice. The findings highlight the role of family logistical support and community physical activity resources in promoting children's FMS, particularly locomotor skills. These results suggest that interventions should not only focus on school-based physical education but also strengthen family support and improve access to community-based physical activity environments. Future research should further examine the mechanisms through which different types of parental support influence specific domains of motor skill development, preferably using longitudinal designs and objective measures. In addition, given the substantial developmental variation within the 6–10 year age range, future studies are encouraged to explore age-specific patterns and mechanisms of fundamental movement skill development in greater detail.

## 6 Conclusion

The FMS of children aged 6–10 years in Macau were generally at a relatively low level and showed developmental imbalance, with locomotor skills outperforming object control skills. Motor skill levels increased progressively with age. Parental logistical support and community resource utilization were significantly associated with children's FMS, particularly locomotor skills, whereas object control skills were mainly influenced by logistical support.

These findings suggest that, in high-density urban contexts, environmental and logistical factors play a more critical role in promoting children's FMS than parental role modeling alone. Accordingly, interventions should not only focus on school-based physical education but also empower parents to act as active facilitators by providing access to resources, enrolling children in structured programs, and creating opportunities for participation.

## Data Availability

The original contributions presented in the study are included in the article/supplementary material, further inquiries can be directed to the corresponding author.
